# Effects of an Alkalizing or Acidizing Diet on High-Intensity Exercise Performance under Normoxic and Hypoxic Conditions in Physically Active Adults: A Randomized, Crossover Trial

**DOI:** 10.3390/nu12030688

**Published:** 2020-03-04

**Authors:** Mirjam Limmer, Juliane Sonntag, Markus de Marées, Petra Platen

**Affiliations:** 1 Department of Sports Medicine & Sports Nutrition, Ruhr-University Bochum, Gesundheitscampus Nord 10, 44801 Bochum, Germany; markus.demarees@rub.de (M.d.M.); petra.platen@rub.de (P.P.); 2 Institute of Outdoor Sports and Environmental Science, German Sport University Cologne, 50933 Cologne, Germany; jule.sonntag@yahoo.de

**Keywords:** acid–base balance, potential renal acid load (PRAL), base–forming nutrition, acid-forming nutrition, moderate simulated altitude, hypoxic chamber, sports nutrition, mountain sport disciplines, anaerobic exercise test

## Abstract

Pre-alkalization caused by dietary supplements such as sodium bicarbonate improves anaerobic exercise performance. However, the influence of a base-forming nutrition on anaerobic performance in hypoxia remains unknown. Herein, we investigated the effects of an alkalizing or acidizing diet on high-intensity performance and associated metabolic parameters in normoxia and hypoxia. In a randomized crossover design, 15 participants (24.5 ± 3.9 years old) performed two trials following four days of either an alkalizing (BASE) or an acidizing (ACID) diet in normoxia. Subsequently, participants performed two trials (BASE; ACID) after 12 h of normobaric hypoxic exposure. Anaerobic exercise performance was assessed using the portable tethered sprint running (PTSR) test. PTSR assessed overall peak force, mean force, and fatigue index. Blood lactate levels, blood gas parameters, heart rate, and rate of perceived exertion were assessed post-PTSR. Urinary pH was analyzed daily. There were no differences between BASE and ACID conditions for any of the PTSR-related parameters. However, urinary pH, blood pH, blood bicarbonate concentration, and base excess were significantly higher in BASE compared with ACID (*p* < 0.001). These findings show a diet-induced increase in blood buffer capacity, represented by blood bicarbonate concentration and base excess. However, diet-induced metabolic changes did not improve PTSR-related anaerobic performance.

## 1. Introduction

Many sport competitions staged at terrestrial altitudes ranging up to 3500 m (e.g., track-and-field, cycling and team sport events, cross-country or alpine ski races, and mountain biking challenges) often require single or multiple bouts of high-intensity, anaerobic exercise performance [1,2,3,4,5]. In addition, although insufficiently investigated to date, several mountaineering disciplines are performed at moderate to high altitudes with high anaerobic demands (e.g., ski touring, and single- or multi-pitch rock, mixed, or ice climbing) [6,7].

However, acute exposure to moderate and high altitudes above 1500 m can impair anaerobic exercise performance [8]. Reduced exercise tolerance above the lactate threshold at altitude is mainly caused by severe disruption to homeostasis resulting from a decline in arterial oxygen saturation (S_a_O_2_) because of reduced oxygen pressure in the ambient and inspired air (P_I_O_2_) [9]. The reduced P_I_O_2_ leads to a decrease in arterial oxygen partial pressure (*P*O_2_) and to hypoxemia, which stimulates the peripheral chemoreceptors to evoke CO_2_ washout [10,11,12]. Concurrently, hyperventilation occurs as a hypoxic ventilatory response during acclimatization to high altitude, while carbon dioxide partial pressure (*P*CO_2_) falls and arterial pH increases according to the Henderson−Hasselbalch equation [10,13,14]. This respiratory alkalosis is subsequently compensated for by the increased renal elimination of bicarbonate ions ([HCO_3_^−^]), which results in a decrease in blood [HCO_3_^−^] and an arterial pH returning to normal [10,11,13]. Blood [HCO_3_^−^] is an essential blood buffer for metabolic acids. During maximal workloads with blood lactate levels up to 15 mmol/L, there is a corresponding decrease in plasma [HCO_3_^−^] [15]. Thus, the resulting decline in [HCO_3_^−^] and blood buffer capacity in the course of altitude adaption may significantly affect anaerobic exercise performance at altitude, particularly above the lactate threshold [10,16,17,18,19].

Regarding anaerobic exercise performance at altitude, several studies have investigated the effects of acute hypoxia on anaerobic performance parameters [20,21,22,23,24,25]. However, there are inconsistent and controversial findings, with reports of either a significant impairment [20,21,22,23,24,25] or unaffected [26,27,28,29] anaerobic exercise performance when exposed to acute hypoxia. Considering the applied study protocols, this inconsistent and often unaffected anaerobic exercise performance may relate to the lack of conformity in the duration of exposure to hypoxic conditions prior to exercise. Metabolic compensation of respiratory alkalosis and the associated [HCO_3_^−^] loss is considered a slow-adapting mechanism. For example, progression after 6 h and completion after 24 h of low to moderate altitude exposure was reported [10,30]. Furthermore, this process was reported to remain incomplete after 24 h of exposure to high altitude, but was completed after some days [10,12,30,31].

However, pre-exercise exposure to hypoxia within these studies investigating anaerobic exercise performance at simulated altitudes mainly ranged between 15 min and 1 h [20,21,22,24,32]. Thus, we suggest that this short exposure to simulated hypoxic conditions does not reflect the time course of renal compensation of hypoxia-induced respiratory alkalosis, and is inappropriate for assessing decreases in anaerobic exercise performance because of the metabolic compensation of respiratory alkalosis and the associated [HCO_3_^−^] loss. Additionally, recent studies reporting no impairment of anaerobic exercise performance mainly used 30-s and 45-s Wingate tests to assess anaerobic exercise performance [26,27,28,29], despite the evidence that short duration, high-intensity exercise performance can be maintained in acute hypoxic conditions because of a shift toward anaerobic metabolism [33,34]. By contrast, power output for tests with continuous or repeated high-intensity exercise longer than 45 s, like the 3-min all-out critical power test and repeated sprints, is often reduced in acute hypoxia [20,21,22,24,32]. Therefore, we propose that performance tests assessing for anaerobic, high-intensity exercise performance in hypoxic conditions should last for more than 45 s.

A number of studies have also examined the positive effects of supplementation with ergogenic aids such as sodium bicarbonate (NaHCO_3_) or dietary nitrate as alkalotic buffers for attenuation of the impaired exercise performance under hypoxic conditions [32,35,36,37,38,39]. Ingestion of NaHCO_3_ increases the [HCO_3_^−^] concentration in extracellular fluids, which leads to an enhanced buffering of hydrogen ([H^+^]) ions [40,41]. This [HCO_3_^−^]-induced increased buffering capacity seems to improve high-intensity exercise performance in normoxia [32,40,42] and hypoxia [32,35,36,37,43,44,45]. A few studies have examined the effect of NaHCO_3_ ingestion on anaerobic exercise performance at altitude. Although some studies found no effect of [HCO_3_^−^] supplementation on the power output of high-intensity exercise at simulated altitudes of 3000 m and 2500 m [46,47], a constant or improved anaerobic exercise performance at simulated altitude compared with anaerobic exercise performance under normoxic conditions was described for participants receiving alkalizing agent supplements prior to exercise [43,44,45]. In addition, positive effects of NaHCO_3_ ingestion under acute moderate normobaric hypoxic conditions during intermittent and repeated high-intensity exercise were reported [32,35,36,37]. In those studies, the authors concluded that NaHCO_3_ ingestion may offer an effective ergogenic strategy to alleviate hypoxia-induced declines in anaerobic exercise performance.

Nevertheless, the effects of an alkalizing or acidizing dietary modification on anaerobic performance under hypoxic conditions has not been investigated to date. Nutrition has a strong impact on acid base balance [48,49,50], above all the urinary acid excretion, intestinal absorption rates of nutrients, and the dietary protein and mineral contents [51,52], which can be quantified via the potential renal acid load (PRAL) [49,53,54,55]. However remaining controversial [56,57], an improvement in anaerobic exercise performance after a low-PRAL (alkalizing) diet for tests with a duration of 60 s to 2 min [48,58,59], as well as an influence on blood and urinary pH, and [HCO_3_^−^], have often been described when following an alkalizing diet [57,59,60].

Overall, these studies suggest that NaHCO_3_ ingestion and the associated [HCO_3_^−^]-induced enhanced buffering capacity may enhance high-intensity anaerobic exercise performance under acute normobaric hypoxic conditions [32,35,36,37,43,44,45]. Additionally, several studies propose that an alkalizing diet can change the blood buffer capacity, which influences high-intensity anaerobic exercise performance in a way similar to NaHCO_3_ ingestion [48,58,59]. However, there are no studies investigating the effects of a low-PRAL (alkalizing) or high-PRAL (acidizing) diet on anaerobic exercise performance after several hours of hypoxic exposure. Therefore, the aim of the present study was to investigate the influence of an alkalizing versus acidizing diet on a single bout of anaerobic exercise performance, maximum capillary blood lactate concentrations, blood gas parameters, heart rate (HR), rating of perceived exertion, and urinary pH (pH_u_) in moderately trained young participants under normoxic conditions and after 12 h of exposure to hypoxia. We hypothesized that an alkalizing diet would enhance extracellular buffering capacity, and thus increase anaerobic exercise performance, under normoxic conditions, and mitigate potential hypoxia-induced declines in anaerobic exercise performance under hypoxic conditions.

## 2. Materials and Methods 

### 2.1. Participants

Sixteen healthy, nonspecifically trained adult volunteers (*n* = 8 men, *n* = 8 women) participated in the present study. One woman withdrew from the study because of a busy schedule. The results presented are for the remaining 15 participants. For men (*n* = 8), the mean (± standard deviation) age was 24.6 ± 4.6 years, the mean height was 180.0 ± 5.6 cm, and the mean body mass was 72.1 ± 5.5 kg, while for women (*n* = 7) these values were 24.4 ± 3.4 years, 167.3 ± 5.9 cm, and 60.9 ± 9.5 kg, respectively. All participants underwent medical screening before entering the study. Participants had to be in good health with no pre-existing altitude illnesses, cardiac or pulmonary conditions, and no musculoskeletal injuries that could interfere with running activities. All participants lived close to sea level, were recreationally active (i.e., practicing various physical activities for 12 h per week), and were familiar with sprinting activities. Exclusion criteria included acute muscular injuries or restrictions, chronic medication intake, alcohol consumption, acute infections, and time spent above 2000 m in the four weeks prior to the investigation. In addition, individuals ingesting any nutritional supplements or following any specific diet in the two months prior to the initiation of the study were excluded. The study was approved by the ethical committee of the German Sport University Cologne in accordance with the Declaration of Helsinki. Participants gave their written informed consent after they had been informed of all experimental procedures and risks.

### 2.2. Experimental Design

**Figure 1:**
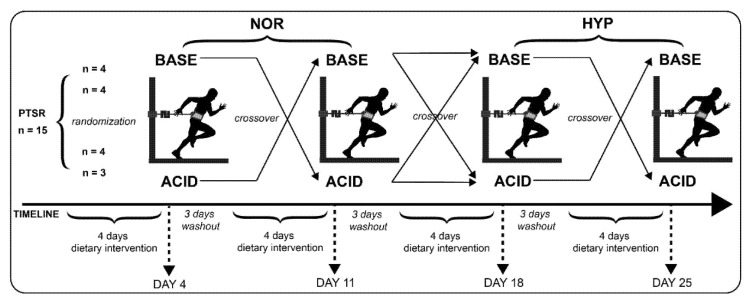
Experimental sequence. NOR = normoxia; HYP = hypoxia; PTSR = portable tethered sprint running test; ACID = acidizing diet; BASE = alkalizing diet.

The present investigation was conceptualized as a randomized, single-blind, counterbalanced crossover trial (Figure 1). Whereas investigators were blinded for treatment assignments, participants needed to be informed about necessary dietary modifications to obtain high or low PRAL values, but not about expected influences of the diets and associated hypotheses. After having ad libitum breakfast, all participants performed four anaerobic performance tests in a laboratory setting at intervals of one week under normoxic (NOR) conditions for the first two weeks and hypoxic (HYP) conditions in weeks 3 and 4. To limit the effects of diurnal variation on the measured variables, the four anaerobic performance test trials were performed at approximately the same time each day. In a randomized order, an acidizing (ACID) or alkalizing (BASE) dietary intervention was followed, resulting in four groups (ABAB, ABBA, BAAB, BABA). This resulted in the four treatment conditions NOR ACID, NOR BASE, HYP ACID, and HYP BASE. Each dietary intervention was maintained for four days [56], followed by three washout-days with an unmodified diet before the next dietary intervention started in a crossover trial. Participants were requested to abstain from strenuous high-intensity exercise and alcohol for 24 h before each trial, and we requested compliance with these instructions before each anaerobic performance test trial.

### 2.3. Dietary Interventions

For the assessment of the daily PRAL values, as well as caloric and fluid intake, participants were asked to document all foods and beverages consumed during the dietary interventions using the Freiburger Nutrition Protocol (Freiburger Ernährungsprotokoll, Nutri-Science GmbH, Hausach, Germany). PRAL values represent a quantification of the effects of ingested nutrients on the acid‒base status [49,53]. The PRAL model is based on the content of proteins, Cl^−^, PO4^3−^, SO4^2−^, Na^+^, K^+^, Ca^2+^, and Mg^2+^ [52] and was calculated as follows: PRAL (mEq/100 g) = 0.49 × protein (g/100 g) + 0.037 × phosphorus (mg/100 g) − 0.021 × potassium (mg/100 g) − 0.026 × magnesium (mg/100 g) − 0.013 × calcium (mg/100 g) [56]. In general, vegetables, fruits, and potatoes have the highest alkalizing potential (low-PRAL nutrients), while meat, cheese, cereal products, and eggs promote systemic acidity (high-PRAL nutrients) [48,54,55]. In addition, a German PRAL food list published by the Institute for Prevention and Nutrition, Ismaning, Germany, [52] and suggestions for recipes were given to the participants to meet the requirements of the alkalizing or acidizing diet. Participants were instructed to make food and amount choices ad libitum based on the respective PRAL values of foods. Following the common recommendations for PRAL-manipulating diets, participants were specifically instructed to eat mainly fruits and vegetables during the alkalizing, low-PRAL diet, combined with energy-dense foods such as starchy vegetables (e.g., potatoes and sweet potatoes), plant sources of fat (e.g., seeds and nuts, avocadoes), and dried fruits (e.g., figs, dates, and raisins). During the acidizing, high-PRAL diet, participants were instructed to eat mainly grains (e.g., oats, bread, pasta), hard cheese (e.g., parmesan), and meats. Nutrients with moderate PRAL values, such as white rice, yogurt, and legumes, were allowed for both dietary trials to ensure an adequate energy intake, especially for the alkalizing diet [48,56]. Based on the daily nutrition protocols, overall fluid intake (∑ fluid), caloric intake (∑ CAL), and overall PRAL sum value (∑ PRAL) were calculated for each participant for the four conditions of NOR ACID, NOR BASE, HYP ACID, and HYP BASE for statistical analyses.

### 2.4. Urinary pH

pH_u_ was determined in spontaneous early morning urine samples (at least 5 mL of urine) using Neutralit® pH-indicator strips pH 5.0–10.0 (Merck, Darmstadt, Germany). pH_u_ was measured on each day of the four-day dietary interventions, and served as a control marker to ensure that the dietary intervention had been implemented successfully [61]. The pH_u_ of day 4 of the dietary intervention, when the portable tethered sprint running (PTSR) test was performed, was used for statistical analyses.

### 2.5. High-Intensity Anaerobic Performance Test

Anaerobic performance was measured using the PTSR test [62]. The PTSR test was chosen because it is simple, requires little space, and does not involve heavy and unwieldy equipment. The PTSR test is suitable for field studies investigating high-intensity exercise performance during altitude sojourns, as well as for the restricted space in hypoxic chambers, and thus allows direct comparability between studies in normobaric and hypobaric hypoxic conditions. For the test, participants ran in one place with a belt round their waist for force measurements. The belt was attached to an inextensible static rope combined in series with a load cell, and fixed to a pillar at 90° to the subject’s waist height. The belt was located at the iliac crest to assure that participants were not hampered to pull maximally against the tether. Before each PTSR test, the participants completed a pretest warm-up, which included 5 min of aerobic exercise and 5 min of coordination and dynamic stretching. ‘Ready’, ‘Set’, and ‘Go’ commands were provided, and the participant performed an all-out sprint for 60 s. At ‘Go’, participants started to sprint at maximum effort and pulled with full force. Study investigators were all PTSR-experienced and provided strong verbal encouragement for the entire test duration to ensure that participants pulled the rope until voluntary exhaustion. Tethered running involves an often unfamiliar moving pattern. Participants who were not familiar with tethered running thus had to perform an additional habituation session prior to the first test trial to assure adequate test implementation of the PTSR test and related physiological responses. Force data were recorded and downloaded to an online PC using a sampling rate of 100 Hz. Overall peak force (PF) and overall mean force (MF) over 60 s were recorded for subsequent analysis. Fatigue level during the PTSR test was assessed by calculating the fatigue index (FI), following the recommended calculations for Wingate tests [63]. HR was recorded as a control parameter throughout the tests using HR monitors (Polar T31; Polar Electro, Kempele, Finland). Thus, HR was measured before and after the PTSR tests. Maximal post-exercise HR after performance tests was used for further analyses. Blood lactate levels were measured in 20-µL capillary blood samples collected from a hyperemized earlobe before and 2, 4, 6, 8, and 10 min after PTSR testing. Blood lactate measurements were carried out directly after each PTSR trial (Biosen S-Line; EKF-diagnostic GmbH, Magdeburg, Germany). The maximum post-exercise lactate concentration (La_max_) occurred mainly between 4 to 6 min after PTSR testing and was used for statistical analyses. Borg’s rating of perceived exertion (RPE) was used to assess subjective perception of effort after each PTSR test [64]. Borg’s RPE was explained to each participant by trained practitioners before the PTSR tests, and was used as a marker for the relationships between subjective measures of exertion and the objectively measured metabolic parameters of blood lactate and blood gas analysis.

### 2.6. Blood Gas Analysis

Capillary blood samples (100 µL) were taken from a hyperemized earlobe before (PRE PTSR) and within 1 min after each PTSR trial (POST PTSR). Blood samples were immediately analyzed for blood gas parameters using a blood gas analyzer (ABL80 FLEX CO-OX; Radiometer, Willich, Germany). *P*O_2_, *P*CO_2_, blood pH (pH_b_), S_a_O_2_, blood [HCO_3_^−^], and base excess (BE) were determined. For HYP trials, additional capillary blood samples were taken before entering the hypoxic chamber (PRE HYP) to assess them for influences on acid‒base balance because of hypoxic conditions.

### 2.7. Anthropometric Characteristics

Body weight was determined with a sliding weight mechanical scale (Seca 709; Seca, Hamburg, Germany). Height was measured (to the nearest 0.1 cm) using the scale-integrated stadiometer.

### 2.8. Hypoxic Conditions

For HYP conditions in weeks 3 and 4 of the experimental period, all test subjects were exposed to a simulated altitude of 3000 m. Altitude was simulated through nitrogen injection (VPSA S325 V16; van Amerongen, Tiel, The Netherlands) in a 65 m^3^ environmental chamber located at sea level. For simulation of an altitude of 3000 m, inspired air consisted of 15.0% O_2_, and the room temperature in the hypoxic chamber was kept at a constant level of 21–23 °C using air conditioning (42 WKR 61; Carrier, Neuss, Germany). For the conditions HYP ACID and HYP BASE, all test subjects were exposed to normobaric hypoxic conditions in two test sessions for 12 h overnight. Participants entered the hypoxic chamber in the evening between 8 p.m. and 9 p.m., and performed the PTSR test the next morning between 8 a.m. and 9 a.m. under hypoxic conditions after having ad libitum breakfast. Participants were asked to perform only quiet and sedentary activities without any further activity specifications during the 12-h stay in the hypoxic chamber.

### 2.9. Statistical Analysis

Data are presented as mean ± standard deviation. All departures from normal distribution were identified using the Shapiro–Wilk test. The effects of treatments on the parameters PF, MF, FI, La_max_*,* HR, RPE, pH_u_, ∑ fluid, ∑ CAL, and ∑ PRAL over time (NOR ACID, NOR BASE, HYP ACID, and HYP BASE) were tested by one-way repeated-measures ANOVA, with sex (male and female) as a between-subject factor. The effects of treatments on the blood gas analysis parameters *P*O_2_, *P*CO_2_, pH_b_, S_a_O_2_, [HCO_3_^−^], and BE over time (NOR ACID PRE PTSR, NOR ACID POST PTSR, NOR BASE PRE PTSR, NOR BASE POST PTSR, PRE HYP ACID, HYP ACID PRE PTSR, HYP ACID POST PTSR, PRE HYP BASE, HYP BASE PRE PTSR, HYP BASE POST PTSR) were tested by one-way repeated-measures ANOVA, with sex (male and female) as a between-subject factor. Violations of the assumption of sphericity were corrected for by Greenhouse–Geisser adjustments. Two-tailed paired *t*-tests were utilized as post hoc tests to indicate significant differences. A Bonferroni procedure was used (p*) to retain an α = 0.05, and the significance level was set at *p* ≤ 0.05 in all comparisons. Effect sizes were calculated using partial η squared (ηp^2^), and were interpreted as small (0.01), medium (0.06), and large (0.14). For post hoc analyses, Cohen’s d (*d*) was used to calculate effect sizes, with 0.2 considered to indicate a small effect, 0.5 a medium effect, and 0.8 a large effect [65].

We also performed stepwise multiple linear regression analyses to elucidate whether the variables ∑ PRAL, ∑ fluid, ∑ CAL, pH_u_, pH_b_ PRE PTSR, [HCO_3_^−^] PRE PTSR, and BE PRE PTSR were predictors of the PTSR-related performance measurements PF, MF, FI, La_max_, and HR. Furthermore, to determine which of the abovementioned variables may predict the PTSR-related measurement of RPE, we performed an ordinal logistic regression analysis.

Finally, we performed an a priori analysis to compute the required sample size for our study, based on a previous study [48], in which a low-PRAL, alkalizing diet resulted in a 21% improvement of anaerobic time to exhaustion (2.56 ± 0.36) compared with a high-PRAL, acidizing diet (2.11 ± 0.31 s). Using an α-level of 0.05, this indicated a sufficient sample size of eight participants to detect the expected changes with a power of at least 0.95. The α-level was set at *p* ≤ 0.05, and all analyses were conducted using statistical software (SPSS v25; IBM Co., Armonk, NY, USA). The free software G*Power was used to calculate the required sample sizes and effect sizes [66].

## 3. Results

### 3.1. Dietary Intervention

**Figure 2:**
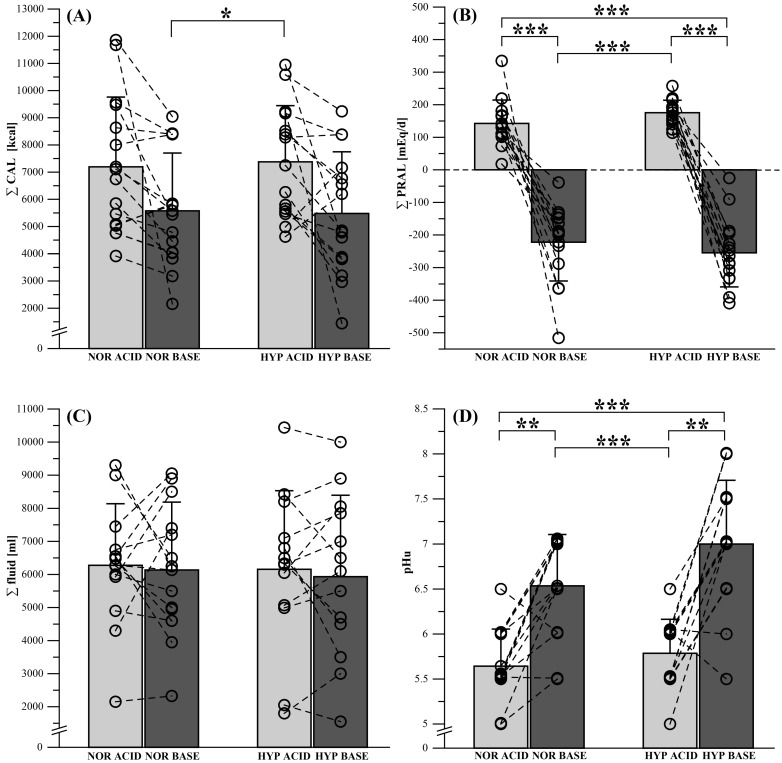
Changes in dietary intervention-related parameters after acidizing (ACID) and alkalinizing (BASE) diet under normoxic (NOR) and hypoxic (HYP) conditions for (**A**) overall caloric intake (∑ CAL), (**B**) potential renal acid load sum value (∑ PRAL), and (**C**) overall fluid intake (∑ fluid), as well as the associated physiological response of (**D**) urinary pH (pH_u_). Data points represent individual values (○). Bar charts are mean ± standard deviation. * *p* ≤ 0.05, ** *p* ≤ 0.01, *** *p* ≤ 0.001. See Section 2. Materials and Methods for further details.

We found significant main effects for ∑ CAL (*p* = 0.014, ηp^2^ = 0.298) and ∑ PRAL (*p* < 0.001, ηp^2^ = 0.888). There was no significant main effect for ∑ fluid (*p* = 0.893, ηp^2^ = 0.009). Post hoc analyses showed significantly lower values in ∑ CAL for NOR BASE (5576.1 ± 2125.4) compared with HYP ACID (7379.5 ± 2066.6 kcal; p* = 0.038, *d* = 0.86), and significantly higher values in ∑ PRAL for NOR ACID (142.6 ± 71.9 mEq/day) compared with NOR BASE (−222.3 ± 118.6 mEq/day; p* < 0.001, *d* = 3.53), HYP ACID (175.6 ± 38.3 mEq/day) compared with HYP BASE (−255.0 ± 103.0 mEq/day; p* < 0.001, *d* = 4.77), HYP ACID compared with NOR BASE (p* < 0.001, *d* = 3.80), and NOR ACID compared with HYP BASE (p* < 0.001, *d* = 4.35) (Figure 2A−C). The participants’ sex had no influence on any of the dietary intervention parameters.

### 3.2. Urinary pH

We found a main effect for pH_u_ (*p* < 0.001, ηp^2^ = 0.655). Post hoc analyses showed significantly lower pH_u_ values for NOR ACID (5.64 ± 0.41) compared with NOR BASE (6.54 ± 0.57; p* = 0.002, *d* = 1.75), HYP ACID (5.79 ± 0.38) compared with HYP BASE (7.0 ± 0.71; * < 0.001, *d* = 1.98), HYP ACID compared with NOR BASE (p* = 0.007, *d* = 1.49), and NOR ACID compared with HYP BASE (p* < 0.001, *d* = 2.21) (Figure 2D). The participants’ sex had no influence on pH_u_ (*p* = 0.376, ηp^2^ = 0.078).

### 3.3. High-Intensity Anaerobic Performance Test

**Figure 3:**
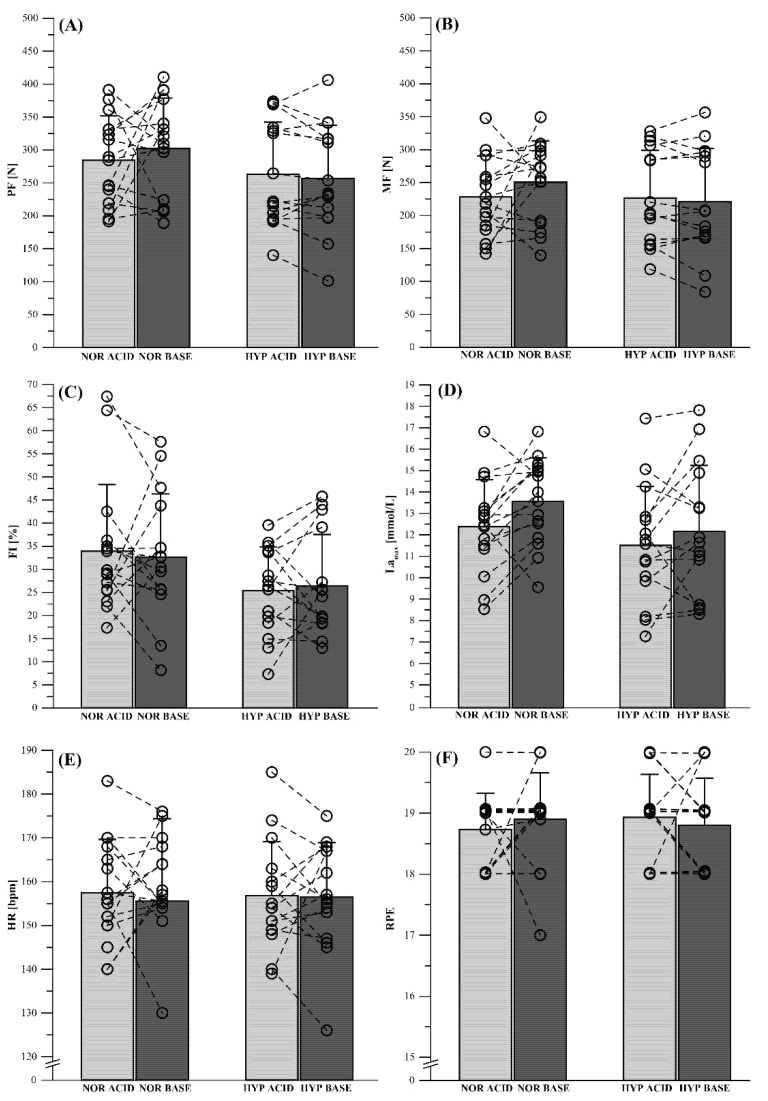
Performance measurements after acidizing (ACID) and alkalinizing (BASE) diet under normoxic (NOR) and hypoxic (HYP) conditions for (**A**) peak force (PF), (**B**) mean force (MF), and (**C**) fatigue index (FI), as well as the associated physiological response (**D**) maximum blood lactate (La_max_), (**E**) heart rate (HR), and (**F**) Borg’s rating of perceived exertion (RPE). Data points represent individual values (○). Bar charts are mean ± standard deviation. See Section 2. Materials and Methods for further details.

Results for all PTSR related parameters are shown in Figure 3. There were no significant main effects in PF (*p* = 0.158, ηp^2^ = 0.132), MF (*p* = 0.300, ηp^2^ = 0.088), and FI (*p* = 0.056, ηp^2^ = 0.174) (Figure 3A–C). However, there was a significant main effect for La_max_ (*p* = 0.011, ηp^2^ = 0.246) (Figure 3D), with significantly lower La_max_ values for male (14.0 ± 1.5 mmol/L) compared with female participants (10.6 ± 0.9 mmol/L; *p* < 0.001). There were also no main effects in HR (*p* = 0.948, ηp^2^ = 0.009) and RPE (*p* = 0.780, ηp^2^ = 0.027) (Figure 3E,F). Additionally, the participants’ sex showed no influence on PTSR-related parameters except for La_max_.

### 3.4. Blood Gas Analysis

There was a significant main effect for *P*O_2_ *(p* < 0.001, ηp^2^ = 0.761), *P*CO_2_ (*p* < 0.001, ηp^2^ = 0.450)_,_ S_a_O_2_ (*p* < 0.001, ηp^2^ = 0.842), pH_b_ (*p* < 0.001, ηp^2^ = 0.941), [HCO_3_^−^] (*p* < 0.001, ηp^2^ = 0.914), and BE (*p* < 0.001, ηp^2^ = 0.931). Significant differences in post hoc tests are shown in Table 1.

Additionally, the participants’ sex showed a significant influence on *P*CO_2_ (*p* = 0.045, ηp^2^ = 0.177) and pH_b_ (*p* = 0.014, ηp^2^ = 0.221). In post hoc analyses, male participants had significantly higher values for *P*CO_2_ compared with female participants in NOR BASE PRE PTSR (male: 41.4 ± 2.9, female: 36.2 ± 1.5 mmHg; p* = 0.010, *d* = 2.26), HYP BASE PRE HYP (male: 42.9 ± 2.5, female: 39.0 ± 1.8 mmHg; p* = 0.040, *d* = 1.85), HYP BASE POST PTSR (male: 44.5 ± 3.0, female: 35.0 ± 5.3 mmHg; p* = 0.020, *d* = 2.19), HYP ACID PRE HYP (male: 42.0 ± 2.8, female: 37.2 ± 2.2 mmHg; p* = 0.030, *d* = 1.88), and HYP ACID POST PTSR (male: 42.0 ± 2.7, female: 33.7 ± 4.9 mmHg; p* = 0.010, *d* = 2.12). For pH_b_, post hoc analyses showed significantly lower values in HYP BASE POST PTSR for male (7.20 ± 0.03) compared with female participants (7.28 ± 0.05; p* = 0.040, *d* = 1.78). The participants’ sex had no significant influence on *P*O_2_ (*p* = 0.220, ηp^2^ = 0.094)_,_ S_a_O_2_ (*p* = 0.131, ηp^2^ = 0.108), [HCO_3_^−^] (*p* = 0.514, ηp^2^ = 0.059), or BE (*p* = 0.160, ηp^2^ = 0.117).

### 3.5. Regression Analyses

**Figure 4:**
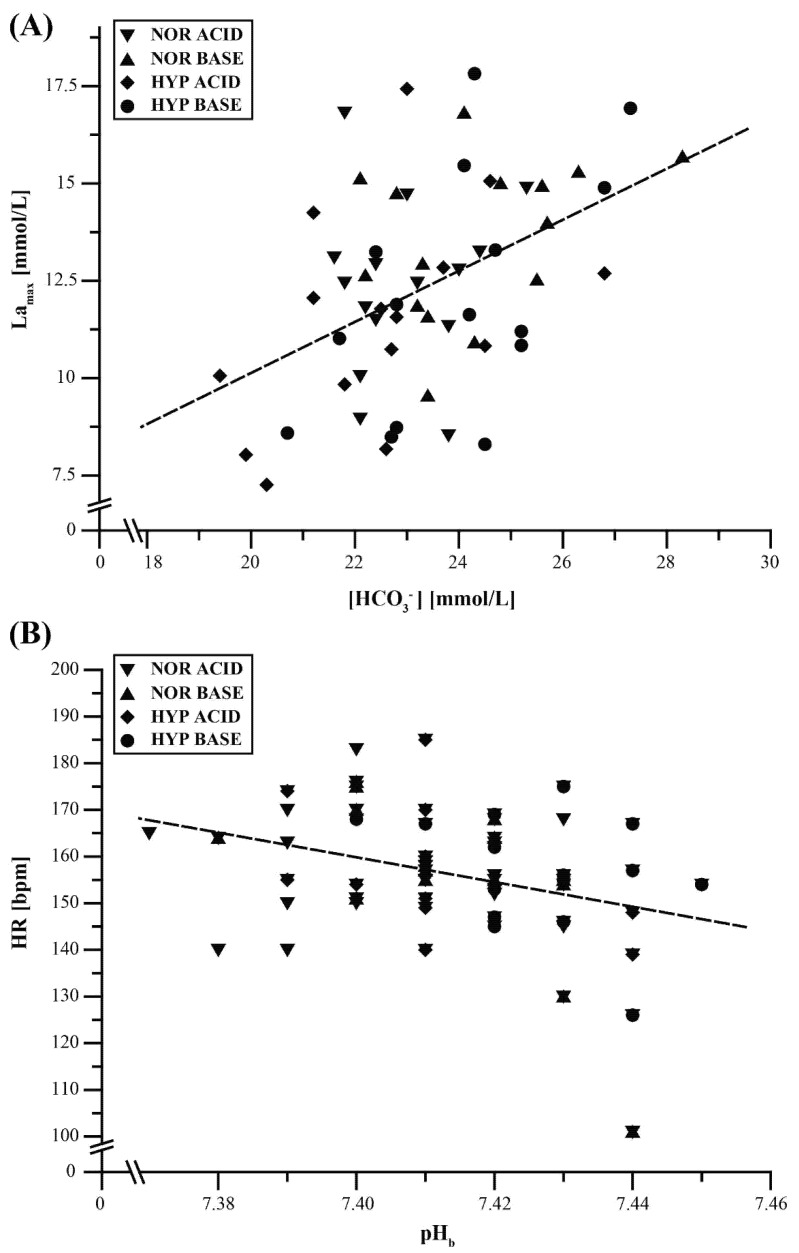
Relationships between PTSR-related (**A**) maximum post-exercise lactate concentration (La_max_) and (**B**) heart rate (HR) and the blood gas measurements before PTSR tests of blood pH (pH_b_ PRE PTSR) and blood bicarbonate ([HCO_3_^−^] PRE PTSR). Data points represent individual values for the four treatment conditions NOR ACID (*n* = 15), NOR BASE (*n* = 15), HYP ACID (*n* = 15), and HYP BASE (*n* = 15). NOR = normoxia, HYP = hypoxia, ACID = acidizing diet, and BASE = alkalinizing diet. See Section 2. Materials and Methods for further details.

Multiple linear regression analyses revealed no relevant predictors for PF, MF, and FI incorporating the variables ∑ PRAL, ∑ fluid, ∑ CAL, pH_u_, pH_b_ PRE PTSR, [HCO_3_^−^] PRE PTSR, and BE PRE PTSR. However, [HCO_3_^−^] PRE PTSR was identified as a significant predictor for La_max_ and pH_b_, while PRE PTSR was identified as a significant predictor for HR, whereas the variables ∑ PRAL, ∑ fluid, ∑ CAL, pH_u_, and BE PRE PTSR did not significantly predict La_max_ and HR. The results of the multiple linear regression analyses on La_max_ and HR are shown in Table 2. Relationships between La_max_ and [HCO_3_^−^] PRE PTSR, as well as HR and pHb PRE PTSR, are shown in Figure 4. Ordinal logistic regression analysis revealed no significant result in the main model fitting for RPE (*χ*^2^ = 0 8.495, *p* = 0.273).

## 4. Discussion

The central aim of this study was to determine the effect of an alkalizing versus acidizing diet on a single bout of high-intensity exercise performance represented by PTSR test performance outputs, maximum capillary blood lactate concentrations, blood gas parameters, HR, rating of perceived exertion, and urinary pH in moderately trained young participants under normoxic conditions and after 12 h of exposure to a simulated altitude of 3000 m above sea level. As such, the main finding of the study was that alkalizing or acidizing diets had no significant influence on PTSR-related performance outputs and associated physiological responses, regardless of a high impact of the dietary interventions on acid‒base balance.

We assumed an adequate implementation of the dietary intervention because overall PRAL values, which represent the acid- or base-forming potential of consumed nutrients, differed significantly between the ACID and BASE conditions. Positive PRAL values reflect an excess of acid-forming, acidizing potential, whereas negative values reflect an excess of base-forming, alkalizing potential [49,53], and we found significantly higher PRAL values for ACID conditions compared with BASE conditions in the present study. Thus, we conclude that our specific instructions for the modification of the participants’ habitual diets were understandable and feasible for the study participants, and that the dietary interventions were able to be included in a daily routine. The conclusion of a successful dietary modification is supported by significantly increased pH_u_ values during the BASE trials, in contrast with the ACID trials. In a recent study, pH_u_ was used as a surrogate marker for a successfully-conducted dietary intervention, and in general, a pH_u_ of ≥ 7.0 was proposed for successful alkalizing diets and ≤ 6.0 for acidizing diets [48,61]. Thus, we assume that the significant increase in pH_u_ values in the present study represents a profound impact on acid‒base balance because of the alkalizing or acidizing diets. In addition, the impact of the dietary interventions on acid‒base balance can be estimated by blood gas analysis parameters, and an increase in [HCO_3_^−^] concentration and elevated pH_b_ are both found after acid‒base manipulation with ergogenic aids such as NaHCO_3_ [40,41].

A few recent studies have suggested that alkalizing diets are unable to produce the same severe effect on acid‒base balance and blood buffering capacity compared with alkalizing ergogenic aids [55,67]. However, the present study showed significantly increased pH_b_, [HCO_3_^−^], and BE values for the BASE condition compared with the ACID conditions, indicating a higher alkalotic state prior to PTSR exercise testing for the BASE trials. It was suggested that metabolic manipulation of the acid‒base balance by NaHCO_3_ ingestion enhances anaerobic exercise performance by increasing the availability of [HCO_3_^−^], thereby strengthening the physiochemical processes of buffering capacity (e.g., stimulation of the lactate/[H^+^] cotransporter) and leading to increased removal of [H^+^] during exercise [32,40,42]. The suggested mechanism underlying the increased [H^+^] efflux from the intracellular to extracellular compartments involves increased removal of [H^+^] from the extracellular buffering systems [40,41], as well as improved protection of intramuscular pH and increased anaerobic energy provision and glycogen utilization [66,68]. Therefore, this leads to the assumption that the higher alkalotic state prior to exercise for the alkalizing diet trials within the present study would result in higher performance outputs in the PTSR trial.

However, despite an apparent influence of the dietary intervention on acid‒base balance parameters and blood buffer capacity, the alkalizing or acidizing diets had no significant effect on PTSR-related performance parameters (PF, MF, and FI), or on the associated physiologic responses of La_max_ and HR. It was previously reported that pre-alkalization prior to exercise had an ergogenic effect for anaerobic exercise performance under normoxic [32,40,42] and hypoxic conditions [32,35,36,37,43,44,45]. However, whereas NaHCO_3_ ingestion is a well-established method for an enhancement of anaerobic performance, the influence of an alkalizing diet on anaerobic exercise performance is still controversially discussed [67] and some investigations reported for less pronounced systemic alkalinity, blood buffer capacity, and an unaffected anaerobic exercise performance after an alkalizing diet [56,57,58]. The present study contributes to this negative assumption as we found no differences in any of the PTSR-related parameters for the ACID or BASE trials under either normoxic or hypoxic conditions. In addition, we assumed that the hypoxia-induced declines in high-intensity, anaerobic exercise performance would appear under normobaric hypoxic conditions, because a significant impairment of anaerobic exercise performance was previously reported [20,21,22,23,24,25], and as a reduced [HCO_3_^−^] concentration and accompanying acidification of extracellular fluids as a consequence of the renal compensation to hypoxia-induced respiratory alkalosis was reported to negatively affect exercise performance at altitude, particularly above the lactate threshold [10,16,17,18,19]. In the present study, we observed significantly reduced *P*O_2_, *P*CO_2_, S_a_O_2_, and [HCO_3_^−^] values after 12 h of exposure to a simulated altitude of 3000 m, indicating a hypoxia-induced respiratory alkalosis. However, a respiratory alkalosis is typically associated with elevated pH_b_ values, which we did not observe. This lack of effect on pH_b_ may be attributed to an ongoing renal compensation of the respiratory alkalosis with subsequent [HCO_3_^−^] loss and restoration of pH_b_ to normal. However, this assumption should be treated with caution because we did not perform hourly acid‒base analysis under hypoxic conditions, and our data do not allow for direct deduction of a [HCO_3_^−^] loss and pH_b_ stabilization based on the lack of a significant increase in pH_b_. Nevertheless, the apparent discrepancies in anaerobic performance outputs and expected diet- or hypoxia-induced changes may be attributable to several factors, as detailed below.

First, we observed a reduced caloric intake for the BASE trials compared with the ACID trials, despite food recommendations for an adequate energy intake during the BASE trial. A caloric deficit during consuming alkalizing diets was previously reported [59], and alkalizing dietary recommendations are presumed for caloric deficits [69]. When consuming alkalizing diets, increasing the consumption of fruits and vegetables, and minimizing consumption of protein (e.g., meats, cheese) and carbohydrate sources (e.g., grains such as bread or pasta) [54], are often suggested to achieve low PRAL values. It is well established that an alkalizing diet makes it difficult to maintain the high caloric intake necessary to meet the high energy demands, and the requirement for dietary protein and carbohydrate sources, reported for sport disciplines with a high anaerobic contribution of energy production [53,54,56]. In particular, an influence of carbohydrate intake on exercise performance with high anaerobic demands was previously reported [70,71]. We provided ad libitum breakfast prior to the PTSR tests but omitted to standardize caloric and carbohydrate intake during breakfast. This may have resulted in individual differences in glucose and glycogen availability and an influence on performance data, and should therefore be considered as a limitation of this investigation. Furthermore, within the present study the caloric deficit and associated reduction in carbohydrate intake under BASE conditions may have mitigated the ergogenic effects of the pre-alkalization. Indeed, consumption of carbohydrate-rich vegetables and fruits, such as fresh and dried fruits, fruit juices, and potatoes, was highly advised to participants when specific instructions for the nutritional modification were explained prior to the test trials [48]. However, in that study, despite a high commitment for implementation of dietary instructions, participants were not able to maintain caloric intake during the BASE trials. Thus, future studies should focus on completion of food diaries, as well as a rigorous control of food intake using daily contact with a dietician to provide specific and individual food suggestions. Additionally, the use of mineral waters rich in [HCO_3_^−^] should be encouraged to simplify achieving an alkalizing diet while maintaining the high-energy diet required for anaerobic exercise performance [53,72,73].

Second, we examined a single bout of anaerobic exercise performance using the PTSR test. The PTSR test was selected because it is simple to setup, requires minimal space, and does not involve heavy and unwieldy equipment. These aspects are important when planning for investigations in altitude field settings. Field investigations assessing exercise performance during mountaineering tours and high-altitude expeditions may require the test equipment to be carried, and the anaerobic testing to be performed in restricted spaces (e.g., a mountain hut). Thus, the PTSR test is one of only a few tests feasible for the investigation of anaerobic exercise performance in altitude field conditions [62]. Other test procedures for the assessment of anaerobic exercise performance in the laboratory or field settings include evaluation of repeated sprint and intermittent sprint performance [74,75,76]. In this context, a recent review suggested that a single sprint of running or cycling activities in the laboratory environment of a hypoxic chamber is unaffected by acute exposure to normobaric hypoxia [77], while larger alterations in sprint outputs were found for repeated sprints or continuous high-intensity exercise lasting longer than 45 s [20,24,32,77]. Additionally, running performance is impaired for single bouts of performance in running distances of 800 m or longer when competing at altitudes above 1000 m [5]. This difference in anaerobic performance outputs may relate to the relatively low contributions of energy from aerobic metabolism required for efforts of short durations (<45 s), and thus the larger anaerobic contribution to the total energy requirement [78]. Aerobic energy availability for sprinting is reduced in oxygen deprived environments [79,80]. Therefore, whereas performance maintenance for single sprints of a short duration in hypoxic conditions is attributed to increased rates of anaerobic energy release that compensate for the reduced aerobic energy production, anaerobic exercise efforts of longer durations or multiple bouts are more affected by hypoxic conditions because of the higher aerobic energy contribution [25,27,77]. We assumed that a test duration of 60 s was sufficient for optimal assessment of continuous exhaustive anaerobic exercise performance as the aerobic/anaerobic energy contribution for a 400-m event usually lasting between 50–70 s was calculated as 41% or 59%, respectively [81]. However, the lack of differences in performance outputs within the present study may among others relate to the applied exercise test protocol, and future investigations may further contribute to the still controversially discussed topic of impaired anaerobic exercise performance in hypoxia using different test protocols including assessments of all-out running for longer durations up to 3 min or repeated sprint performance.

The theory of strong ion difference (SID) may also explain the unexpected lack of an ergogenic effect of the alkalizing diet [82]. Our findings were based on the Henderson–Hasselbalch approach, which presumes that blood pH is determined by changes in [H^+^] and [HCO_3_^−^]. However, the contrasting SID theory incorporates intracellular and extracellular ions, and describes the difference between the concentrations of strong cations (sodium, potassium, calcium, and magnesium) and strong anions (lactate and chloride). The SID was also suggested to affect muscle performance by altering intracellular or extracellular pH because of an independent effect on blood pH [82]. The SID approach may therefore explain the increase in pre-PTSR [HCO_3_^−^] and BE for the BASE conditions with simultaneously persistent pH_b_ in normoxia. Thus, acidizing dietary interventions may have had a positive impact on intracellular and extracellular ions, and following muscle performance, regardless of changes in [H^+^] and [HCO_3_^−^]. However, this conclusion should be interpreted with caution because no SID measurements were conducted in the present study. Thus, future studies are required to examine the influence of changes in the SID on anaerobic exercise performance under hypoxic conditions.

A low statistical test power is a common study limitation used to explain a lack of expected effects. Although an a priori analysis was performed prior to the present investigation, the number of 15 participants is still a small sample size and may result in small test power for statistical analyses. Thus, we reported our effect sizes, and found medium to large effect sizes for ∑ CAL, PRAL, pH_u_, PF, MF, FI, La_max_, *P*O_2_, *P*CO_2_, S_a_O_2_, pH_b_, [HCO_3_^−^], and BE, ranging between ηp^2^ = 0.088 and ηp^2^ = 0.941. These data indicate sufficient testing power for analyzing the effect of an alkalizing or acidizing dietary intervention and normobaric hypoxic conditions on these parameters. Thus, we conclude that our sample size of 15 participants was sufficient to detect possible differences in the investigated dietary, PTSR-related, and BGA-related parameters, and to exclude a type 2 error within our interpretation.

## 5. Conclusions

We provide novel data on the effects of an alkalizing or acidizing dietary intervention on anaerobic exercise performance under normoxic or hypoxic conditions after 12 h of exposure to a simulated altitude of 3000 m. Our principle finding was that dietary intervention significantly increased the blood buffer capacity, represented by pre-exercise [HCO_3_^−^] and BE values, but did not affect PTSR-related exercise performance outputs or associated physiologic parameters. A higher alkalotic state of the acid–base balance prior to exercise under hypoxic conditions is often associated with higher anaerobic performance outputs and higher maximum blood lactate values after high-intensity exercise in normoxic and hypoxic conditions. Explanations for the apparent lack of any ergogenic effect of pre-alkalization caused by an alkalizing diet include a reduced caloric intake for the BASE trials compared with the ACID trials, the duration of the 60-s portable tethered sprint test and the associated energy contributions, and possible changes in intracellular and extracellular ions other than [H^+^] and [HCO_3_^−^].

## Figures and Tables

**Table 1:** Portable tethered sprint running test (PTSR)-related blood gas parameters after acidizing or alkalinizing diet under normoxic and hypoxic conditions.

			*P*O_2_ [mmHg]	*P*CO_2_ [mmHg]	S_a_O_2_ [%]	pH_b_	[HCO_3_^−^] [mmol/L]	BE [mmol/L]
**N** **O** **R**	ACID	PRE PTSR	85.7 ± 7.6 #	37.6 ± 2.2	98.4 ± 1.1 #	7.40 ± 0.02	22.9 ± 1.1	−0.7 ± 1.3
		POST PTSR	91.5 ± 9.7 #	42.2 ± 5.0	96.9 ± 1.4 #	7.20 ± 0.05	15.7 ± 2.0	−11.4 ± 2.6
	BASE	PRE PTSR	85.6 ± 4.0 #	38.9 ± 3.5	98.3 ± 0.9 #	7.41 ± 0.02	24.3 ± 1.7 *	0.1 ± 1.3
		POST PTSR	89.9 ± 7.4 #	43.1 ± 5.5	97.0 ± 13 #	7.23 ± 0.04	17.2 ± 2.1 *	−10.8 ± 2.8
**H** **Y** **P**	ACID	PRE HYP	90.1 ± 9.0 #	39.8 ± 3.5 #	98.6 ± 0.9 #	7.39 ± 0.02	23.7 ± 1.5 #	−0.6 ± 1.2
		PRE PTSR	67.8 ± 4.3	36.2 ± 3.8	94.4 ± 1.3	7.41 ± 0.02	22.5 ± 2.0	−1.2 ± 1.5
		POST PTSR	72.8 ± 5.9	38.1 ± 5.7	91.5 ± 2.9	7.22 ± 0.06	15.1 ± 1.8	−12.0 ± 2.4
	BASE	PRE HYP	90.3 ± 7.9 #	41.1 ± 2.9 #	98.5 ± 0.6 #	7.41 ± 0.01	25.5 ± 1.6 #*	1.2 ± 1.3 *
		PRE PTSR	66.3 ± 4.6	37.1 ± 3.3	93.3 ± 1.4	7.43 ± 0.01 *	24.0 ± 1.8	0.4 ± 1.4
		POST PTSR	70.3 ± 5.6	40.1 ± 6.4	90.8 ± 2.1	7.24 ± 0.06	16.5 ± 2.0	−10.3 ± 2.5

Note: Data are presented as mean ± standard deviation. *P*O_2_ = oxygen partial pressure; *P*CO_2_ = carbon dioxide partial pressure; S_a_O_2_ = oxygen saturation; pH_b_ = blood pH value; [HCO_3_^−^] = blood bicarbonate concentration; BE = base excess; ACID = acidizing diet; BASE = alkalinizing diet; NOR = normoxia, HYP = hypoxia, PRE PTSR = pre-PTSR values; POST PTSR = post-PTSR values. For further details see Section 2. Materials and Methods * *p* < 0.05 vs. ACID, # *p* < 0.05 vs. HYP. For *p*-values see Section 3. Results.

**Table 2:** Linear multiple regression analysis on portable tethered sprint running test (PTSR)-related maximum post-exercise lactate concentration (La_max_) and heart rate (HR).

	Predictor Variable	R^2^	Corrected R^2^	F	p	Standardized β	T	p
**La_max_**	Model	0.200	0.184	12.746	0.001 *			
	∑ PRAL					0.073	0.539	0.592
	∑ Fluid					0.030	0.241	0.811
	∑ CAL					0.145	1.163	0.250
	pH_u_					−0.212	−1.689	0.097
	pH_b_ PRE PTSR					−0.224	−1.823	0.074
	*[HCO3-] PRE PTSR*					*0.447*	*3.570*	0.001 *
	BE PRE PTSR					−0.304	−1.021	0.312
**HR**	Model	0.091	0.073	5.084	0.028 *			
	∑ PRAL					−0.176	−1.255	0.215
	∑ fluid					0.065	0.480	0.633
	∑ CAL					0.168	1.265	0.212
	pH_u_					0.089	0.603	0.550
	*pH_b_ PRE PTSR*					*−0.301*	*−2.255*	0.028 *
	[HCO_3_^−^] PRE PTSR					0.172	1.294	0.202
	BE PRE PTSR					0.183	1.367	0.178

Note: Linear multiple regression on La_max_ and HR in response to potential renal acid load sum value (∑ PRAL), overall fluid intake (∑ fluid), overall caloric intake (∑ CAL), urinary pH (pH_u_), baseline blood pH value (pH_b_ PRE PTSR), baseline blood [HCO_3_^−^] ([HCO_3_^−^] PRE PTSR), and baseline BE (BE PRE PTSR); (*n* = 60). * *p* ≤ 0.05.

## Dataset

All datasets generated for this study are available from the figshare repository database (doi:10.6084/m9.figshare.11534547).
